# Challenges, stressors, and resilience resources experienced by older black women in Rural South Carolina throughout the COVID-19 pandemic

**DOI:** 10.1371/journal.pone.0342512

**Published:** 2026-02-24

**Authors:** Arielle N’Diaye, Camryn Garrett, Sara Wilcox, Bankole Olatosi, Xiaoming Li, Shan Qiao

**Affiliations:** 1 Department of Health Promotion, Education, and Behavior, Arnold School of Public Health, University of South Carolina, Columbia, South Carolina, United States of America; 2 South Carolina SmartState Center for Healthcare Quality, Arnold School of Public Health, University of South Carolina, Columbia, South Carolina, United States of America; 3 Department of Exercise Science, Arnold School of Public Health, University of South Carolina, Columbia, South Carolina, United States of America; 4 Prevention Research Center, Arnold School of Public Health, University of South Carolina, Columbia, South Carolina, United States of America; 5 Department of Health Services, Policy, and Management, Arnold School of Public Health, University of South Carolina, Columbia, South Carolina, United States of America; 6 The Big Data Health Science Center, University of South Carolina, Columbia, South Carolina, United States of America; Durham University, UNITED KINGDOM OF GREAT BRITAIN AND NORTHERN IRELAND

## Abstract

**Background:**

Resilience has been established as a protective factor against mental health challenges (e.g., anxiety, depression). From an intersectional perspective, the stressors experienced by Black women living in rural communities are likely to have been exacerbated during the COVID-19 pandemic. This study aims to identify the challenges and stressors experienced and the resilience resources available to and relied upon by rural, Black women, during the pandemic.

**Methods:**

Individual, in-depth, structured interviews (n = 24) were conducted among Black women living in rural South Carolina, recruited by local community health workers, from January to April of 2024. All interviews were recorded, uploaded to a password-protected online drive, transcribed using Otter.ai, and verified for verbatim transcription. The data were analyzed using an abductive approach where the data were inductively coded following a thematic analysis approach and were then deductively situated within a socioecological model.

**Results:**

Participants shared challenges and stressors (i.e., anxiety, depression, isolation, grief, visitation limitations, employment changes, increased cost of living, structural and systemic inequity, and limited access to mental healthcare) experienced throughout the pandemic. Resilience resources were also identified at the individual (i.e., religion and faith, self-care), interpersonal (i.e., generational knowledge of preparedness, social connection), organizational (i.e., workplace and religious institution pandemic policy adaptation), community (i.e., rural community norms, trusted community messengers, food and supply drives), and structural levels (i.e., emergency SNAP enrollment, stimulus checks) of the socioecological model.

**Conclusions:**

The present results suggest that interventions, designed to prepare and respond to public health emergencies, should leverage and amplify existing community-led resilience resources, thereby adequately tailoring efforts and ensuring they meet the needs identified by community members.

## Introduction

In the United States, the COVID-19 pandemic exacerbated existing racial health disparities [1,2]. For example, people of color experienced higher rates of COVID-19 infection, higher levels of hospitalization for COVID-19, and worse COVID-19 health outcomes (i.e., mortality, disease severity) [1,2]. In 2023, Black Americans were 1.1 times more likely than white Americans to experience COVID-19 infection, were 2 times more likely to be hospitalized for the virus, and were 1.6 times more likely to experience COVID-specific mortality [3]. Disparities in COVID-19 outcomes may be attributed to the increased risk, as related to pre-existing health conditions (e.g., cardiovascular disease, diabetes, obesity), that stems from the upstream effects of structural racism [4,5]. For example, differential occupational exposures exist as a result of structural racism, where Black populations are more likely to be essential workers and exposed to COVID-19, due to higher levels of interpersonal contact in workplaces that may not adopt or enforce COVID-19 protective policies (e.g., telework options, paid sick leave) or provide personal protective measures (e.g., masks, social distancing) [6]. When considered by urbanization, rural areas, compared to urban areas, experienced, on average, lower levels of engagement with COVID-19 prevention practices (e.g., masking, vaccination), higher rates of COVID-specific mortality, and higher incidence rates [7–9]. Additionally, as related to COVID-19 vaccination, in 2022 it was estimated that 75% of urban dwelling individuals had received their first dose of the COVID-19 vaccine compared to 58.5% of individuals in rural settings [8].

Given the presence and effects of racial and geographic health disparities, Black women in rural areas faced a variety of COVID-19 pandemic-specific stressors. Pandemic-related stressors have been found to stem from disruptions to daily routines, changes in financial status, fears around themselves or family members contracting COVID-19, and mental health challenges that could arise as a result of COVID-19 specific challenges and stressors (e.g., depression, isolation, anxiety) [10]. Additionally, stressors were observed as stemming from the inability to engage with others in person and the reduction or closure of social support resources (e.g., churches) [11]. Further, scholars have concluded that Black women faced unique vulnerabilities to stressors and subsequent adverse health outcomes during the pandemic as a result of structural racism and gendered expectations (e.g., serving as caretakers, parenting and assisting with virtual schooling while working) [10,12,13].

In light of these disparities and vulnerabilities, scholars assert that further research is needed to understand the presence and manifestation of resilience factors among Black women in rural communities during the COVID-19 pandemic [14]. The concept of resilience is traditionally defined, in the literature, as the ability to navigate adverse experiences and challenges [15,16]. Within a scoping review of resilience among Black women, a multitude of definitions have been found to have a varying focus, such as on individual abilities to overcome adverse experiences and challenges, having an adaptive personality, having access to community and social support, or resilience as fostered through resource accessibility and quality [16].

A growing body of literature describes resilience as a dynamic, multidimensional process. Notably, a systematic review of the literature describes the highly contextual nature of resilience (e.g., regional, geographic, SES contexts), particularly as influenced by factors at various socioecological levels [15]. As informed by the Model of Structural Resilience to Minority Stress, which extends the Minority Stress Model beyond gender and sexual minorities to racial and ethnic minorities, resilience is conceptualized beyond the individual to consider the breadth of factors influential on resilience processes, ranging from the individual to the macro- and chronosystems [17].

The existing literature identifies knowledge gaps in terms of the need to identify the resilience resources used by Black women in rural communities, and to explore how these resources were used in response to daily challenges during the pandemic [5]. For example, the literature has called for qualitative research, using individual or focus group data collection methods, to better understand and contextualize both individual and community resilience within rural Black communities during the COVID-19 pandemic [18]. Specifically, research is needed to understand the role of historic and emergent stressors as well as resilience resources in the wake of the COVID-19 pandemic for application in future unprecedented times, including global stressors such as emergent infectious diseases [5]. Therefore, to advance our understanding of Black women’s resilience, particularly in rural settings, there is a need to explore the dynamic, process-oriented experiences of individual coping and contextual resources across all levels of the socioecological model. In efforts to address these literature gaps, the present work utilizes an assets-based orientation to consider the factors and resources that facilitate resilience beyond the level of the individual, with the aims to explore the following research questions: 1) What challenges did Black women living in rural South Carolina face during the pandemic? and 2) What resilience resources were used to cope with these challenges?

## Methods

### Participants and Recruitment

Eligible participants were those aged 18 years or older, who self-identified as a Black woman, and who lived in rural South Carolina throughout the COVID-19 pandemic. Participants were purposively sampled from 6 rural counties across South Carolina by community health workers (CHWs), through a partnership with the statewide CHW association. CHWs who were engaged in the present research activities were required to complete a training led by the statewide CHW association and the research team. The training covered topics related to the project purpose, foundations of community-based research, privacy and confidentiality, interview methodology, ethics, equity, and data quality and validity. All CHWs signed a confidentiality agreement in which they agreed to keep all information confidential to protect the privacy of all participants. As one component of the training, the CHWs reviewed the interview guide and provided feedback that was used to refine and tailor the questions, ensuring its applicability to the target study population. Upon the completion of the training and signature to the confidentiality agreement sponsored by the statewide CHW association, CHWs began participant recruitment. CHWs relied on their professional and social networks, within the communities in which they live and work, to recruit potential participants. As reinforced through the training, CHWs were instructed to exclude their friends and family from their recruitment and interview efforts.

All study activities were conducted in accordance with the Declaration of Helsinki and the Belmont Report. The study protocol was reviewed and deemed exempt by the Institutional Review Board at the University of South Carolina (Pro00123957).

### Data Collection

All interviews were conducted by trained CHWs. As part of a community-academic partnership, these CHWs were relied upon for qualitative interviewing due to their familiarity with the communities in which they live and work [19]. The interviews followed a structured interview guide that was developed in a collaboration between the research team and the statewide CHW association. The interview guide covered domains such as COVID-19 experiences, social support systems, coping strategies, and experiences of stigma and discrimination. Participants were asked questions such as, “What aspects of the pandemic have challenged your mental health?”, “Can you tell me about the support systems you have?”, “How can or do those around you support you?”, “Can you describe the main causes of stress for you?”, and “What do you typically do to deal with this stress?” Participants were not provided a specific time period to consider but were rather asked about their experiences throughout the pandemic.

Multiple interview modalities were anticipated (i.e., in-person, telephone, Zoom) to ensure that the interviews could be conducted in a rural setting, where internet connectivity may be limited, as well as to ensure the interviews could be at a time and location that was convenient and preferred by the participants. Although there are slight differences in the advantages and disadvantages between interview modalities, online and telephone interviewing have been deemed somewhat comparable and acceptable within certain settings, including rural settings [20,21].

Participants were provided with an invitation to participate, which operated as the form of informed consent and described the study’s purpose, confidentiality procedures, risks, benefits, and the voluntary nature of participation. All interviews were audio recorded and then transcribed using Otter.ai [22]. Each transcript was verified, line-by-line, by a member of the research team to ensure it was transcribed verbatim and deidentified. The interviews ranged from 10 to 40 minutes in duration and were conducted between January and April of 2024. Information power was evaluated to determine the end point of recruitment [23]. Information power can be understood as a principle that considers the relevance of the provided information to weigh the number of participants needed as dependent on the study aims, sample specificity, theoretical underpinnings, quality of interview dialogue, and the planned analytical strategy [23].

### Qualitative Approach and Research Paradigm

The present study employed a thematic analysis approach and was theoretically underpinned by the socioecological model of health, which situates individuals within their broader contexts [24]. Theoretically, the socioecological model was selected because it provides a lens through which to conceptualize how access to resources may have shaped the challenges and stressors experienced and resources relied upon at various levels (i.e., individual, interpersonal, organizational, community, structural). Onto-epistemologically, this study is situated within a critical-constructivist paradigm where we maintain the worldview that multiple realities exist that are socio-historically constructed, that people are the experts of their own experiences, and that the knowledge produced by people is socio-historically bound [25]. The onto-epistemological orientation was selected because our analytical approach conceptualizes the experiences of the women featured within this study in relation to both the past and present.

### Data Analysis

The transcripts were analyzed following an abductive thematic analysis approach. Whereby transcripts were inductively analyzed following Braun and Clarke’s (2006) process of data familiarization, initial code generation, theme searching, and theme review, and were then deductively analyzed following their process of theme definition and naming [26]. The data were coded by two authors (CG, AN) and were consistently reviewed by another (SQ). The data analysis process began with the compilation of the code pool, which was then topically organized. The codes were then sorted into categories and subcategories, which then comprised the themes and subthemes. An analysis matrix was then drafted to detail the substantiation between the subcategories, categories, subthemes, and themes. The analysis matrix of the study findings was reviewed and discussed amongst the research team to obtain consensus on the study results, and was then adapted into a figure, informed by the socioecological model, to illustrate the emergent themes. A reflexive journal and an audit trail were maintained throughout the data analysis process. Exemplary quotes are used to illustrate emergent themes. The present findings are reported in accordance with the Standards for Reporting Qualitative Research Checklist [27].

### Reflexivity and Researcher Characteristics

Within this study, the authors and community partners offer perspectives from a variety of social positionings relevant to the work. All authors reside in the southeastern United States. One author resides rurally (CG), while the others reside in urban settings (AN, SW, BO, XL, SQ). Two of the authors are white American women, one author is a Chinese woman, one author is a Chinese man, one author is a Nigerian man, and one is a Black American woman. All authors have previous graduate-level training in qualitative research methods. Among the co-authors, the co-first authors are doctoral students with graduate and undergraduate degrees in public health and sociology. Other co-authors hold doctorate degrees.

## Results

A total of 24 participants were included in the study (Table 1). Based on the inclusion criteria, all participants identified as Black women. The average age of the participants was 60.08 (SD = 16.06) years and ranged from 20 to 82, where 75% of participants (n = 18) were 50 or older. A majority of participants attended some college or held a higher education degree (n = 16, 66.67%). Although many participants chose not to share their annual income (n = 11, 45.83%), others reported earning less than $25,000 USD (n = 2, 8.33%), $25,000 to $49,999 (n = 4, 16.67%), or $50,000 to $100,000 (n = 7, 29.17%). Almost half of the participants indicated ever having COVID-19 (n = 11, 45.83%).

**Table 1:** Overview of Participant Demographics.

Demographic	N (%)
**Gender**; Women	24 (100%)
**Age**, *mean (SD)*	60.08 (16.06)
**Race/ Ethnicity**; Black	24 (100%)
**Rurality**; Rural Dwelling	23 (95.83%)
**Education**	
Less than Highschool	2 (8.33%)
Highschool or Equivalent	6 (25%)
Some College	6 (25%)
Associates	2 (8.33%)
Bachelors	4 (16.67%)
Graduate	4 (16.67%)
**Annual Income**	
< $24,999	2 (8.33%)
$25,000 to $49,999	4 (16.67%)
$50,000 to $100,000	7 (29.17%)
Prefer not to say	11 (45.83%)
**Ever had COVID-19**	11 (45.83%)

Although not captured within the aims and primary findings of the study, the language used to discuss mental health challenges and stressors, by both the researchers and the participants, is pertinent to the present work. While some participants named the mental health challenges they experienced (i.e., anxiety, depression, isolation), others did not. For example, one participant described what appear to be symptoms of depression, but did not refer to it as such when she described her experiences with difficulty sleeping, forgetfulness, and stress (Participant 4). Similarly, another participant described experiencing nervousness, being scared, having heightened cortisol, and having a racing mind with repetitive thoughts, which may be symptoms of anxiety, but were not named as such (Participant 8). When alternative language was used to describe mental challenges (e.g., bottled up, more to myself, wallowing, nervousness) the research team considered how to best categorize participant experiences. In some instances, participants academically named their mental health challenges once probed by the interviewer. Therefore, it is essential to consider the findings given that the research team imparted their academic understandings to classify participant experiences within conventional definitions of mental health challenges.

**Fig 1:**
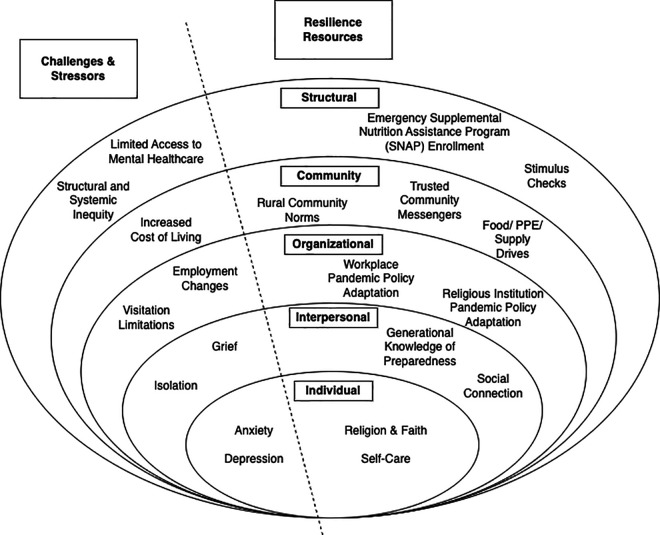
Primary Findings of Challenges, Stressors, and Resilience Resources of Black Women Living in Rural South Carolina during the COVID-19 Pandemic.

Overall, as depicted within Fig 1, participants described challenges, stressors, and resilience resources at all levels of the socioecological model (i.e., individual, interpersonal, organizational, community, and structural). At the individual level, participants experienced anxiety and depression and relied on the individual resilience resources of their faith and self-care practices. Interpersonally, participants described challenging experiences with grief and isolation alongside relying on the resilience resources of social connection and a generational knowledge of preparedness. At the organizational level, participants shared challenges and stressors stemming from social distancing policies and recommendations (e.g., visitors not allowed inside nursing homes or hospitals) and employment changes while simultaneously relying on workplace and religious organization pandemic policy adaptations as resilience resources. Within their communities, participants described the increased cost of living as a primary stressor and their rural community norms, trusted community messengers, and supply drives (i.e., food, PPE, household supplies) as resilience resources. Finally, at the structural level, structural and systemic inequity and limited access to mental healthcare were identified as challenges and stressors and emergency Supplemental Nutritional Assistance Program (SNAP) enrollment and stimulus checks were described as resilience resources.

### Individual Level

At the individual level, participants described facing challenges of COVID-induced anxiety and depression. For example, one participant said, *“My anxiety! I’d never had anxiety until COVID. I never, ever, ever... well, I’m not gonna say I haven’t had, I never dealt with anxiety until COVID came.”* (Participant 2). Participants also described experiencing health anxiety related to fears of a COVID-19 infection and related complications or morbidity. One participant described the interplay between her COVID-related health and social anxiety when she explained, *“Just from like, not wanting to be sick, not wanting anything to happen because the numbers were, were like getting like really high, like people were dying… I just had like really, really bad anxiety just like scared somebody’s gonna get sick. I’m gonna get sick. Somebody’s gonna die, because it had been a lot of it happening. So, it had me a little scared to be out and interacting.”* (Participant 3). In addition to social and health anxiety, some participants also reported experiencing anxiety related to their caregiving roles. Aiding in the management of a loved one’s chronic condition, one participant highlighted the anxiety she felt as a caregiver throughout COVID, when she said:

*“But I had a [loved one], I have a [loved one], who was actually, at the time, going through cancer chemotherapy. That, I was [their] resource for getting [them] to [their] appointments and back home because living in these areas, we have to travel like an hour to get to the chemo for [them] to take… And [their]... hold on. That was a lot. Like right now. I’m having anxiety even remembering because I couldn’t show my anxiety. I had to keep calm to keep [them] calm. So that was a lot. Yeah… I felt guilty having the feelings that I was having. And then after [they] leveled off, and we got through all of what we had to go through, I was so burned out. And I’ve never even talked about that.”* (Participant 11).

Additionally, one participant mentioned experiencing depression, as a result of isolation, when she said, *“It caused, it definitely had an impact on depression. So, I was, I was definitely depressed, couldn’t get out.”* (Participant 1). Despite experiencing depression, one participant shared, *“I’m really good during a crisis. And even with me, from what I observe now, as being depressed, I was still functional enough to accomplish some really major goals, and to serve the community in a way that allowed major opportunities to exist.”* (Participant 7).

Alongside the individual level challenges and stressors identified, participants also described their faith and self-care practices as resilience resources. For example, one participant shared:

*“Well, I guess that goes back to the spiritual side with church. We learn when you’re dealing with something, you give it to God, you pray over it. So, if you prayed over something, you’re stressing, then you may as well not pray about it, you know, so that that knowledge came long before COVID came. So that was, I guess that was my tool… I don’t stress over a lot of stuff because if I can’t do anything about it, I give it to God.”* (Participant 17).

Further, another participant expressed gratitude for and highlighted the importance of her faith, particularly as related to her role as a caregiver, when she mentioned:

*“But you know, through it all. I just pray to God and ask him to give me the strength to get through it all, to do the things that, that I needed to do… I thanked him for getting me through what I had gone through with my [family]. And I just pray that I can live each day to the fullest, and to do the things that I wasn’t able to do before. And to live my life to the best of my ability.”* (Participant 14).

Participants also described various ways they engage in and prioritize self-care (e.g., meditation, relaxation, connecting with others, connecting with nature, engaging in hobbies). One participant described her self-care routine as *“Prayer, music, um calling my, my daughter and talking to my grandbaby. So yeah, and I find that it does help when, you know, I have somebody to kind of share that with or to forget about it and kind of reconnect with somebody that, that means a lot to me.”* (Participant 8). Another participant mentioned, *“So, so I give her love, she gives me love. I do line dancing. I’ve gotten involved in doing that. And so, I just, I just stay busy. I stay busy. And that, that helps me and I’m able to get back into my yard and start doing some of the things that I haven’t been able to do when my [loved one] was sick.”* (Participant 14). Similarly, another participant explained, *“I like to walk a lot. I walk a lot, and it helps me, you know, decompress. I like to be outside in my garden. That helps me a lot. I don’t let a lot of things get to me. I’m at that point in my life to where it’s just not the worry. God is my strength. And that’s what I stand on.”* (Participant 22).

### Interpersonal Level

Participants described interpersonal level challenges and stressors, during the pandemic, as primarily related to their grief and isolation. For instance, one participant mentioned, *“It’s, you know how at times you just don’t feel good? You don’t, you’re, there are times when, you know like, I’m still, I’m still going through my grief.”* (Participant 6). Further, another participant described:

*“I got to the point; I’ve gotten to the point now where I don’t even like to stay home by myself. From 2020 up until last year, I lost so many family, all of it wasn’t to COVID, but I lost so many family members… and it’s taken a toll on me… and all I can do was just think. Thinking that the death that was coming so close to each other, like three in less than a year. It really took a toll on me…”* (Participant 18).

Relatedly, participants repeatedly cited the impact of the COVID-19 pandemic on their experiences of social disconnection. Participants often described the negative effects of social distancing and isolation alongside their understanding that such measures were necessary to remain safe and healthy throughout the pandemic. Referring to the need to social distance to prevent the spread of COVID-19, one participant shared, *“It’s just, it just is a different style of living now. You know, because I feel isolated. You know what I’m saying? … That’s, that’s what I’m talking about, the isolation. You want to go and visit family and friends, your children, your grandchildren. But you know, you have to be careful because not everybody is wearing a mask.”* (Participant 9). Another participant mentioned, within the context of living alone, *“I would think everybody was affected emotionally because we were confined to our homes. We couldn’t go anywhere, couldn’t, was limited what you can do.”* (Participant 17).

In contrast to the stressors of grief and isolation, social connection was highlighted as a primary interpersonal resilience resource. Most participants detailed the importance of connecting with others and a few described the role of social media as a tool for connection, such as when one participant described:

*“You know, we’ve joined groups where we, it’s broadened our thinking, I know for sure. And I, and for me, that that also is a great coping, for me, because being a part of different groups and communities socially, on social media, and then we, I can use some of those same thoughts and ideas and strategies in my life here that they’re using in other places, to, you know, help me to live or basically do what I need to do daily.”* (Participant 11).

Another participant described her role in fostering connection as an interpersonal resilience resource, throughout the pandemic, when she shared, *“They just real small group that I tried to bring together to discuss the different challenges that you had to go through trying to overcome the pandemic was, was good because we would sing and dance and play games and things like that.”* (Participant 4).

Additionally, multiple participants described the importance of a generational knowledge of preparedness in the context of the COVID-19 pandemic, citing their upbringing as the source of their knowledge. For example, one participant described:

*“Yes ma’am. My grandmother always say, ‘Don’t, don’t be caught, hit with, half the stuff. Don’t be caught with nothing in your pocket.’ She always said, ‘You make sure your home is taken care of first and foremost.’ And that was everything that we had to learn from everything we had to go through. As being a Black, young mother, I had to make sure that was in the house. And we were taught thataway.” (Participant 13).*

Similarly, another participant mentioned, *“… because I live alone, I, you know, I’m pretty much self-sufficient. So basically, what I had in the house, I needed, that I could get groceries, so anything outside of that, I mean, I would say I was prepared.”* (Participant 17). Further, a participant explained, *“That that’s the type of person I am. I try to be prepared. And you know, during COVID, you couldn’t come in my house. It was, you just could not without a mask.”* (Participant 22). Another participant described her preparedness in terms of hygiene and as a form of adherence to pandemic prevention recommendations when she said, *“Other than that, no, I wouldn’t, didn’t never, I was never stressed out because, like, we was taught to make sure our bodies, our hands, you know, to make sure we was clean. You know, you get up in the morning, you wash up. Before you go in the refrigerator, you wash your hands. We had been taught that, thataway, you know. We had to be clean.”* (Participant 13). Further, in addition to physical preparedness, some participants described their spiritual preparedness as a resilience resource, such as when one participant said:

*“Oh, that’s easy. That is so easy. Because, you know what I always tell myself? That 'God has prepared me for the next hardship.’ And that’s exactly what I say. And it’s so true. Because, you know, they say he ain’t going to give you more than you can handle. And at the time you think you can’t handle it. And then you get through it, and you look back and you say, ‘That wasn’t as hard as I thought.’ And then you come to the next hurdle. So, and you say to yourself, ‘If I got through that, I can get through this.’”* (Participant 9).

### Organizational Level

At the organizational level, participants identified challenges and stressors from organizational pandemic-related policies that limited visitation within healthcare settings (e.g., hospitals, nursing homes) as well as changes to the workplace. In reference to her husband, for whom she was the primary caretaker, one participant mentioned, “*I wasn’t allowed to go in to see him.*” (Participant 14). Another participant described:

*“She was dehydrated. So that made her be like, kind of wacky, just not coherent. And we could not go into the emergency room, we had to literally sit out in the parking lot. And the only way that we actually was able to communicate with someone was when we would go and go to the, to the door of the outside door, you couldn’t even go into the emergency room, that the nurses were outside. So, we would just have to wait until the doctor actually came out and talked to us.”* (Participant 22).

Additionally, another participant described the importance of video-calls facilitated by the hospital Chaplain as a way in which she was able to see her loved one, despite organizational policies prohibiting them, when she shared:

“*Oh man. Horrible, because we wasn’t, this was at the time when the hospital was on lockdown. Couldn’t go see her. Couldn’t touch her. But they did have a chapel that came in. And we grew fond to him because he allowed us to the video with her. Even though he wasn’t supposed to, but he did it anyway*.” (Participant 19).

Despite the average, older age of the participants, some identified employment changes as organizational challenges and stressors throughout the pandemic. For example, one participant described, “*It was harder to go, go to work basically. I worked in a nursing home so it, you know, mask were worn and people would call out of work every day. So, my workflow did increase. I worked 14-hour days more often.*” (Participant 16). Another participant explained challenges adapting to COVID-19 prevention measures in her workplace when she said:

“*But, um, we had a lot of clients, people with disabilities that I used to work with, intellectual disabilities, that it was so hard trying to keep masks on them. Because they didn’t understand. So, it was like, you have a population of people over here that don’t understand about the mask, and then some people would wear it across their mouth and not over their nose. You know, and then we didn’t always wear it properly. So it was, it was a lot trying to get them to understand about the importance of the mask and training them every day. And you know, sometimes people will bring them in with no mask. So, it was, it was really mentally exhausting. Mentally exhausting a lot of days.*” (Participant 15).

Although changes to the workplace and employment were sometimes identified as a challenge and stressor, workplace pandemic policy adaptation was also described as an organizational resilience resource. For instance, one participant described an adaptation adopted by her employer, where employees were able to share their leave time with one another and how it served as a resource, when she explained, *“And also with my job, with my, my time, my job does participate or well, I [was] participating in a share program, which is for time, for leave time. So, once I exhausted my leave time, I was eligible to be into a share program.”* (Participant 1).

Additionally, religious institution pandemic policy adaptation served as a resilience resource that allowed participants to continue to practice their religion at a time when in-person services were limited. For example, one participant described the modalities offered when she said, “*They had church service on Zoom, online, live.*” (Participant 21). Another participant described attending religious services outdoors when she shared, “*Well I had my church, we had service outside. So, we got, we sat outside of our cars or in our cars, we had service. And then soon as service was over everybody got in their cars and departed because of the fact that we were having [it] outside because of COVID.*” (Participant 17).

### Community Level

An increased cost of living was the primary challenge or stressor identified at the community level. Participants described persistent economic challenges throughout the pandemic, of which many participants mentioned as a result of pandemic-related inflation. For example, one participant described the costs associated with pandemic prevention measures when they said:

*“Because the pandemic you know, was declared over, so you couldn’t just really walk into some place and get a free test. So, I had to pay for the testing. Which was crazy. And my insurance, or the insurance that I had, you know, I won’t be able to get reimbursed until I have to turn in, you know, paperwork and all that stuff to even get reimbursed. So, I had to pay out of pocket every time I went in for those four tests, which ran me about 100 plus dollars. Crazy.”* (Participant 11).

Another participant shared her experience with economic challenges during the pandemic and praised food drives as a community resilience resource when she said:

“*And prices were steadily going up, housing's going up. So, I mean, who could afford it? I mean, where’s the American dream?... The support from the community was, thank God for the food drives, because I did, I did get food myself. Especially with the vegetables and things they really came. That, that was a blessing, because vegetables and fruit is expensive*.” (Participant 9).

An additional participant shared her role in sharing resources gathered from local drives with her community when she explained:

*“Yes, I used some, but my thing is this. If I feel like I got a good bit of food, even if I wanted some and I know there’s a family out there that doesn’t have, I give it to them because I got enough for me. I feel like for somebody that don’t have enough, why keep all this for me when there’s a family out there hungry. That’s the way I look at it. That’s the way I was brought up.”* (Participant 18).

Participants also mentioned receiving personal protective equipment (PPE) and other household supplies (e.g., cleaning supplies) from community drives and loved ones, such as when one participant noted:

“*You know, so we, you know, would get cleaning products if we were all, you know, we would help one another with cleaning products. And if we got PP[E], you know, masks and gloves and things like that, you know, they would share those things, especially because they knew I was out in the community. If they were at a giveaway, they would get extra, so that they could share with me, and I truly appreciate it.*” (Participant 11).

Similarly, another participant described, *“My [loved one]…he was working at the store, and he would get me, when it was kind of hard to get, like that Lysol and stuff.”* (Participant 18).

In addition to the physical resilience resources that were shared within the participants’ rural communities, community members who were viewed and served as trusted messengers were also identified as a community resilience resource. One participant described:

*“I felt better because I knew I could get the correct answers for what’s going on. Because a lot of people didn’t want to believe what they heard. They didn’t, they didn’t believe what was really going on. And I knew that if I talked to my [family member], who’s been a nurse for about 40 years, my [family member] who is a physician assistant, and my [family member] who is a nurse practitioner, and also I talked to my, my, family doctor, too, because I don’t like listening to things, people out in the streets who don’t know what they’re talking about. I like to go to the sources.”* (Participant 6).

Broadly, rural community norms of looking out for and taking care of neighbors were described as a resilience resource during the pandemic. One participant described the support she received from her neighbor when she said:

*“And I have a neighbor that lives across the road from me. He always comes and he take my trash out, pull my garbage out for me, and he’ll bring the cans back. And if he don’t see me during the daytime, he gone call and check on me. If anybody come here, whether I see them or not, if I’m not home, he’s like, more like a watch person, for me.”* (Participant 18).

Similarly, another participant described the support she receives from and gives to her community when she shared:

*“I had people checking on me, I would check on other people, some of my elderly, one of my elderly friends, well, you know, older, and my neighbor who’s down the street, she and I tried to do some running around with them in the first, you know, in the beginning, that they wouldn’t have to come out. You know, I told him, I said, ‘Well, just tell me what you need. When I, before I come from work, you know, I got my mask on…’ you know, we tried to make sure we had everything in place. And I tried to do some things for them so they wouldn’t be, because of that, because of their age, trying to help you know, help other people during this time.”* (Participant 15).

### Structural Level

Participants shared a few instances in which structural and systemic inequity acted as a challenge and stressor during the pandemic. For example, one participant described her experience:

*“Um, I told my truth in the room, that, you know, they really didn’t see the impact of COVID, even though, even though it was very prevalent and real, and the news was covering it. But because it wasn’t affecting them, because they had the vaccine, and their families had it already, they weren’t seeing what the other community, like myself, was seeing, because we already mis-, had a mistrust of the government, pushing medicine or vaccines and knowing, you know, the Tuskegee experiments, things that have happened in African American history, that made us distrustful of what they were trying to do. It definitely impacted our community even more, because we weren’t trying to even go get it. And they had already got it. So, they weren’t seeing the same impact… It really affected me because I’m like, okay, everyone in here, that doesn’t look like me, has already been vaccinated. They weren’t worried about COVID, or dying, or seeing the, the disparities that I was seeing in my community, because it didn’t affect them. And they let it be known. You know, they were kind of shocked that I was being affected the way I was because they didn’t see it for themselves. So that was that was eye opening for me.”* (Participant 12).

Another participant described receiving differential treatment when seeking healthcare when she said, *“When I went to go get tested for COVID, when I first had it. Um, a lot of, especially of the opposite race, like the Caucasian woman. She got back. She got tested before me even though I was there before she arrived…The doctor that took my test, she, she dismissed it at first as a common cold.”* (Participant 16). Additionally, related to her physical ability, one participant shared her experiences when she said, *“I mean, nobody talks to me anyway, because they see [a mobility aid] and they think that I am mentally challenged. And so, they will ask the person with me things like ‘What is my birthday?’ and ‘What is my social security number?’ And I just answer because that’s ridiculous. [laughter].”* (Participant 7).

Furthermore, limited access to mental healthcare was identified by some participants as a challenge and stressor throughout the pandemic. For example, one participant described:

*“It was very stressful because I felt that there was limited access to some of the health services, what I needed, which was mental health, was one of them… I felt like the resources just wasn’t available for the area I was living in. They weren’t really, they weren’t necessarily targeted for African Americans. So, I felt that, really at that point, I felt that the healthcare system failed me…Yes, as an African American female, the healthcare system did fail me… I’ve actually, I’ve actually taken a Mental Health First Aid class. I am now a Mental Health First Aid instructor. So, learning more about mental health has helped tremendously. But it took me initiating, wanting to learn more, do more, and move forward.”* (Participant 1).

Where some participants identified challenges accessing mental healthcare during the pandemic, those who were able to engage in care described its benefits, such as when one participant shared:

*“So you know, it’s easier to kind of, you know, just talk about some things, there’s some things you want to talk about, that you don’t want judgment. First of all, you know, and you don’t want people looking at you differently. What type of person that I am, I’m a very, you know, in the leadership role that I have, you know, as far as work is concerned, as far as school is concerned, you know, some people tend to take weakness and take advantage of it. Or they see, you know, they see something and consider it as a weakness where it’s not a weakness. So, you have to, you know, save face. To save face is not good all the time. So, that’s what my counselor is there for, so I could kind of talk to her.”* (Participant 2).

At the structural level, participants identified financial governmental assistance as resilience resource throughout the pandemic. For instance, emergency Supplemental Nutrition Assistance Program (SNAP) enrollment served as a resilience resource amid the rising cost of living during the pandemic, such as when one participant shared, *“I actually applied and got emergency stamps for, for COVID so that I could get food and stuff. So, I got support like that.”* (Participant 3). Relatedly, participants mentioned the positive assistance provided through stimulus checks, such as when a participant said, *“Oh, I loved stimulus checks, even though it affected my taxes. But the temporary money was really great.”* (Participant 7).

## Discussion

Overall, the present work outlines both the challenges and stressors experienced as well as the resilience resources used by Black women living in rural South Carolina throughout the COVID-19 pandemic. Whereas previous research has identified common challenges and stressors throughout the pandemic (e.g., anxiety, depression, post-traumatic stress disorder, insomnia), across populations, this study contributes to the existing literature as it identifies structural and systemic inequity as a stressor of Black women living in rural South Carolina during the pandemic [28]. Additionally, as the rural context may alter the presence and accessibility of resilience resources, our study identifies resilience resources, across all socioecological levels, that were relied upon, within a rural context, throughout the pandemic.

A notable, spontaneous finding within the present study relates to slight variations between the terminology used by participants, or researchers, to describe mental health challenges (i.e., both acute and long-term). Existing literature has previously identified a similar phenomenon where the language used to describe mental health has been found to differ between clinical terminology and descriptors used by Black populations [29–31]. Scholars have identified sociocultural variations in definitions and presentations of mental health conditions that are not captured in the current clinical guidelines, and the subsequent disregard of the overlapping structures and systems of oppression underpinning the determinants of mental health for this population (e.g., racism, classism) [31,32]. Further, between the binary genders (i.e., men and women), amongst Black populations, differences in symptom expression have been identified but are not currently reflected within mental health assessment tools [32]. Use of mental health assessments that are not culturally applicable may lead to underdiagnosis, misdiagnosis, undertreatment, and mistreatment of mental health conditions among Black populations [32].

Within the present study, religion and spirituality were largely identified as resilience resources through which participants were able to name, understand, and cope with the mental health challenges experienced (e.g., faith, prayer, handing it over to God). Within the literature, religious organizations have been identified as cultural community centers and as spaces where individuals can gather and cope with the psychological and social effects of oppression and stressors (e.g., racism), particularly in times of hardship and crisis [11]. For example, spirituality has been found to facilitate coping among Black breast cancer survivors, such as through the joy, courage, meaning, and protection it provided when feeling fearful, isolated, or financially burdened, even in times such as COVID when access to in-person religious activities may have been limited [33]. Additionally, the literature has found that Black patients previously diagnosed with COVID and those experiencing long COVID relied on their religion to persevere [34,35]. Due to the role of religion and spirituality, the literature has called for mental health interventions, that are tailored to Black populations, to incorporate religion and spirituality to enhance their efficacy [36,37]. Within the present study, participants described their religion as a means of coping with mental health stressors and challenges, mentioned certain mental health challenges as a potential weakness of faith, and identified the role of their spiritual leaders as counselors. In addition to the benefits of spirituality on resilience, there may also exist a tension in perceptions of mental health challenges and care that require further investigation for interventions that are culturally acceptable and appropriate [36–38]. While religion and spirituality play a significant role in facilitating resilience, it is necessary to simultaneously consider a variety of perceptions of and responses to mental health challenges and care that exist within broader contexts that may limit likelihood to access to mental healthcare among marginalized populations, particularly during times of crisis (e.g., COVID-19) [39].

Of note in our study was the pride participants took in their preparedness for withstanding times of uncertainty, including the COVID-19 pandemic and related recommendations and policies (e.g., shelter-in-place orders). Existing literature characterizes the rural south’s sparse population density and limited access to healthcare and social service providers as vulnerabilities that could contribute to observed disparities in COVID-19 health outcomes [14]. The present results contribute to the existing literature by illustrating how Black women living in rural communities adapted, particularly within the context of the COVID-19 pandemic, to fill gaps in formal resource provision as a community, through their social support networks. For example, in areas characterized by limited healthcare access, study participants identified certain community members (e.g., healthcare providers) and church leaders as trusted messengers. This finding is robustly reflected within existing scholarship on the church as a resilience resource within Black communities whereby scholars have identified the church as a consistent knowledge hub and have observed that, historically, religious leaders maintain an influential role in their communities [40]. Further research is necessary to investigate, in more depth, the role of the church as a resilience resource in times of crisis (e.g., COVID-19, natural disaster) and the church’s organizational resilience. Additionally, as the role of the church and religion was discussed within the present study in the context of an older population, future research should prioritize methods that can capture a range of generational experiences [11,41].

When considering the presence and role of resilience resources, it is necessary to also consider the upstream factors (e.g., social determinants of health, policies) that influence access to and quality of resources. For instance, although the present study identified personal preparedness as a key individual resource and described the ways in which communities distributed resources (e.g., social support, personal protective equipment), a lack of resources available to rural communities and lack of tailoring of resources to Black women were also identified as barriers. The structural and social determinants of health can be understood, in the present context, to affect both the access to and quality of key resilience resources and as tied to subsequent health inequities and disparities [42]. Additionally, the influence of the socio-structural determinants of health must be considered from an intersectional perspective as rural Black women are likely to experience key determinants affecting racial minorities (e.g., systemic racism, discrimination), women (e.g., patriarchy, gender norms), and rural communities (e.g., healthcare access and quality, internet connectivity, Medicaid expansion) simultaneously and multiplicatively.

The present study is characterized by several, primary strengths and limitations. One key strength of the present study is its assets-based orientation, which allowed us to gain a nuanced understanding of both the challenges and resilience resources that shaped the lived experiences of Black women in rural South Carolina during the COVID-19 pandemic. The use of an audit trail, thick description, multiple coders, and consulting with community members to develop the interview guide enhanced the credibility and dependability of our study. These findings may be transferable to other rural, deep south settings but are primarily limited to older populations and the population specified in the inclusion criteria. Capturing the perspectives and experiences of an older population makes a significant contribution to the literature. Additionally, having trained CHWs conduct the interviews strengthened the study, as the CHWs were engrained in the rural counties included in the present study, but also acted as limitation because the newly trained CHWs refrained from probing (e.g., asking follow-up questions, seeking clarification) and conducted interviews that were shorter in duration than is common in interviews conducted by academic researchers. As the data in the present study was collected in 2024, and participants were asked to reflect on their experiences throughout the pandemic, recall bias may operate as a limitation.

Based on these findings and limitations, future research should seek to explore how resilience, in theory (e.g., conceptualization) and practice may differ across contexts (e.g., age, urbanization). Similarly, further research should consider how generations may differ in their conceptualization and perception of key drivers of health inequities and disparities (e.g., SDOH, stigma, discrimination) to better tailor public health efforts to target population needs. Based on the study findings, future research is needed to understand how resilience resources may vary across generations of Black women residing within rural communities. In considering the impact of language, there is a further need to explore the distinctions and potential effects of using language in academia that differs from historically marginalized populations, such as to name nervousness as anxiety, as doing so may obscure or misrepresent realities, may perpetuate harmful assumptions, and may mis-medicalize experiences.

Regarding public health implications, the results of the present study suggest that interventions, designed to prepare and respond to public health emergencies, leverage and amplify existing community-led resilience resources, thereby adequately tailoring efforts and ensuring they meet the needs identified by community members. For instance, employing a community-based participatory research framework in future work could leverage the generational knowledge and pride in preparedness, found in the present study, in expanding ongoing mutual aid efforts that equip communities with physical resilience resources. Further work, operating from an assets-based understanding of resilience as a process beyond the individual should consider the potential for structural interventions, designed to meet the needs identified by the community, that utilize established and trusted structures and organizations within their community, thereby considering intervention sustainability and strengthening the existing resilience environment.

## Conclusion

This study explored both the challenges experienced by Black women in rural communities during the COVID-19 pandemic and the resilience resources used to cope with these challenges. Following an assets-oriented inquiry, our study adds to the existing literature by demonstrating how individuals experiencing the COVID-19 pandemic at the intersections of race, gender, and rurality leveraged community, family, and faith-based resources to navigate pandemic-specific challenges.

## Supporting information

S1 File Analysis Matrix of Key Themes. (DOCX)
