# Supplementary material for: Challenges, stressors, and resilience resources experienced by older black women in Rural South Carolina throughout the COVID-19 pandemic
Source: PLoS One. 2026 Feb 24;21(2):e0342512. doi: 10.1371/journal.pone.0342512 (PMC12931777; doi:10.1371/journal.pone.0342512)
Supplement: S1 File — (DOCX) [file pone.0342512.s001.docx]

**Analysis Matrix of Key Themes**

| **Subcategories** | **Categories** | **Subthemes** | **Themes** | **Research Questions** |
| --- | --- | --- | --- | --- |
| Generalized anxiety | Anxiety | Individual Level | Stressors and challenges were experienced across the socioecological model. | What are the challenges experienced by Black women living in rural South Carolina throughout the COVID-19 pandemic? |
| Social anxiety |  |  |  |  |
| Health anxiety |  |  |  |  |
| Caregiver anxiety |  |  |  |  |
| Sadness | Depression |  |  |  |
| Feel a weight |  |  |  |  |
| Social disconnection | Isolation | Interpersonal  Level |  |  |
| Social distancing |  |  |  |  |
| Withdrawing |  |  |  |  |
| Lost family members | Grief |  |  |  |
| Lost members of their social network |  |  |  |  |
| Grief for their community |  |  |  |  |
| Unable to attend medical appointments with loved ones | Visitation Limitations | Organizational  Level |  |  |
| Unable to see loved ones in hospitals long-term healthcare settings |  |  |  |  |
| Unemployment | Employment Changes |  |  |  |
| Changing responsibilities at work |  |  |  |  |
| Changes to work hours |  |  |  |  |
| Limited leave time |  |  |  |  |
| Financial strain | Increased Cost of Living | Community Level |  |  |
| Changing financial situations |  |  |  |  |
| Food costs |  |  |  |  |
| PPE costs |  |  |  |  |
| Stigma and discrimination | Structural and Systemic Inequity | Structural  Level |  |  |
| Racial inequities |  |  |  |  |
| Gender inequities |  |  |  |  |
| Disease status and ability inequities |  |  |  |  |
| Inaccessible mental healthcare | Limited Access to Mental Healthcare |  |  |  |
| Lack of tailoring |  |  |  |  |
|  | | | | |
| Prayer as a tool | Religion and Faith | Individual Level | Resilience resources were identified across the socioecological model. | What are the resilience resources used by Black women living in rural South Carolina throughout the COVID-19 pandemic? |
| Gratitude for faith |  |  |  |  |
| Religion as stress-relief |  |  |  |  |
| Engaging in hobbies | Self-Care Practices |  |  |  |
| Maintaining a busy schedule |  |  |  |  |
| Meditation and Relaxation |  |  |  |  |
| Changing thought patterns |  |  |  |  |
| Physical activity |  |  |  |  |
| Household activities |  |  |  |  |
| Connecting with nature |  |  |  |  |
| Cleaning and hygiene practices | Generational Knowledge of Preparedness | Interpersonal Level |  |  |
| Maintaining resources in the home |  |  |  |  |
| Spiritual preparedness |  |  |  |  |
| Social support | Social Connection |  |  |  |
| Maintaining relationships online |  |  |  |  |
| Importance of others in their life |  |  |  |  |
| Remote work | Workplace Pandemic Policy Adaptation | Organizational Level |  |  |
| Choice in scheduling |  |  |  |  |
| Time-share programs |  |  |  |  |
| Socially distanced religious services | Religious Institution Pandemic Policy Adaptation |  |  |  |
| Online religious services |  |  |  |  |
| Looking out for your neighbors | Rural Community Norms | Community Level |  |  |
| Checking on community members |  |  |  |  |
| Sharing resources within the community |  |  |  |  |
| HCP community members | Trusted Community messengers |  |  |  |
| Religious leaders |  |  |  |  |
| Food distribution | Resources drives |  |  |  |
| PPE distribution |  |  |  |  |
| Cleaning supply distribution |  |  |  |  |
| Emergency Supplemental Nutrition Assistance Program (SNAP) enrollment | Governmental Assistance | Structural Level |  |  |
| Stimulus checks |  |  |  |  |
